# Supplementary material for: Divergent discourse between protests and counter-protests: #BlackLivesMatter and #AllLivesMatter
Source: PLoS One. 2018 Apr 18;13(4):e0195644. doi: 10.1371/journal.pone.0195644 (PMC5906010; doi:10.1371/journal.pone.0195644)
Supplement: S1 Table — (PDF) [file pone.0195644.s020.pdf]

**#BlackLivesMatter**	Nodes	% Original Nodes	Edges	Clustering
Nov. 24–Nov. 30, 2014	270	8.21%	526	0.0598
Dec. 3–Dec. 9, 2014	389	6.86%	912	0.0645
Dec. 20–Dec. 26, 2014	209	6.66%	439	0.1537
Feb. 8–Feb. 14, 2015	76	5.16%	107	0.1744
Apr. 4–Apr. 10, 2015	85	4.38%	121	0.0893
Apr. 26–May 2, 2015	266	6.30%	547	0.1030
Jun. 17–Jun. 23, 2015	176	6.70%	269	0.07690
Jul. 21–Jul. 27, 2015	233	6.67%	435	0.0926
**#AllLivesMatter**				
Nov. 24–Nov. 30, 2014	29	6.43%	40	0.1200
Dec. 3–Dec. 9, 2014	31	3.92%	51	0.2801
Dec. 20–Dec. 26, 2014	49	4.72%	86	0.2626
Feb. 8–Feb. 14, 2015	24	4.67%	30	0.1521
Apr. 4–Apr. 10, 2015	8	2.15%	7	0.0000
Apr. 26–May 2, 2015	41	4.45%	71	0.1967
Jun. 17–Jun. 23, 2015	23	4.77%	33	0.4909
Jul. 21–Jul. 27, 2015	45	5.82%	61	0.1010
