# Supplementary material for: Divergent discourse between protests and counter-protests: #BlackLivesMatter and #AllLivesMatter
Source: PLoS One. 2018 Apr 18;13(4):e0195644. doi: 10.1371/journal.pone.0195644 (PMC5906010; doi:10.1371/journal.pone.0195644)
Supplement: S2 Table — (PDF) [file pone.0195644.s021.pdf]

**#BlackLivesMatter**	Nodes	% Original Nodes	Edges	Clustering
Nov. 24–Nov. 30, 2014	298	9.07%	583	0.0590
Dec. 3–Dec. 9, 2014	444	7.83%	1025	0.0595
Dec. 20–Dec. 26, 2014	227	7.24%	485	0.1494
Feb. 8–Feb. 14, 2015	82	5.57%	119	0.1946
Apr. 4–Apr. 10, 2015	99	5.10%	139	0.0905
Apr. 26–May 2, 2015	294	6.96%	607	0.0976
Jun. 17–Jun. 23, 2015	190	7.23%	305	0.0795
Jul. 21–Jul. 27, 2015	250	7.15%	475	0.0898
**#AllLivesMatter**				
Nov. 24–Nov. 30, 2014	29	6.43%	40	0.1200
Dec. 3–Dec. 9, 2014	37	4.68%	62	0.2456
Dec. 20–Dec. 26, 2014	54	5.21%	94	0.2500
Feb. 8–Feb. 14, 2015	24	4.67%	30	0.1521
Apr. 4–Apr. 10, 2015	10	2.69%	9	0.0000
Apr. 26–May 2, 2015	44	4.77%	76	0.1944
Jun. 17–Jun. 23, 2015	23	4.77%	33	0.4909
Jul. 21–Jul. 27, 2015	47	6.08%	66	0.1151
