# Supplementary material for: Divergent discourse between protests and counter-protests: #BlackLivesMatter and #AllLivesMatter
Source: PLoS One. 2018 Apr 18;13(4):e0195644. doi: 10.1371/journal.pone.0195644 (PMC5906010; doi:10.1371/journal.pone.0195644)
Supplement: S3 Table — Some #AllLivesMatter topic networks have less than 10 top nodes due to the relatively small size of the networks. For example, in the period of April 4th, the #AllLivesMatter network consists of #blacklivesmatter as a hub with six edges connecting it to six other hashtags. Thus, #blacklivesmatter is the only node visited when calculating paths for betweenness. (PDF) [file pone.0195644.s022.pdf]

	**#BlackLivesMatter**		**#AllLivesMatter**	
	Top Hashtags	Betweenness	Top Hashtags	Betweenness
Nov. 24–Nov. 30, 2014	1. ferguson	0.8356	1. blacklivesmatter	0.6116
	2. mikebrown	0.1584	2. ferguson	0.4550
	3. standup	0.0845	3. policebrutality	0.1733
	4. tamirrice	0.0835	4. mikebrown	0.1566
	5. truth	0.0804	5. fergusondecision	0.1566
	6. fergusondecision	0.7935		
	7. arrestdarrenwilson	0.0716		
	8. justiceformikebrown	.0.0704		
	9. shutitdownatl	0.0645		
	10. alllivesmatter	0.0488		
Dec. 3–Dec. 9, 2014	1. icantbreathe	0.4434	1. blacklivesmatter	0.5153
	2. ericgarner	0.4126	2. shutitdown	0.3490
	3. ferguson	0.2706	3. ferguson	0.2827
	4. civilrights	0.0952	4. ericgarner	0.2750
	5. shutitdown	0.0939	5. policebrutality	0.2045
	6. lebronjames	0.0823	6. tcot	0.1908
	7. rip	0.0654	7. icantbreathe	0.1693
	8. cantbreathe	0.0624	8. miami	0.0667
	9. mikebrown	0.0611	9. crimingwhilewhite	0.0667
	10. nojusticenopeace	0.0514	10. handsupdontshoot	0.0114
Dec. 20–Dec. 26, 2014	1. icantbreathe	0.5229	1. nypd	0.4884
	2. ferguson	0.2938	2. policelivesmatter	0.3307
	3. antoniomartin	0.2105	3. blacklivesmatter	0.3064
	4. shutitdown	0.1575	4. icantbreathe	0.2461
	5. ny	0.1174	5. bluelivesmatter	0.2012
	6. handsupdontshoot	0.1012	6. ferguson	0.1833
	7. racism	0.0948	7. handsupdontshoot	0.1679
	8. obama	0.9411	8. nyc	0.1461
	9. mlk	0.0702	9. nojusticenopeace	0.0987
	10. justice	0.0640	10. ericgarner	0.0910
Feb. 8–Feb. 14, 2015	1. blackhistorymonth	0.6504	1. muslimlivesmatter	0.5661
	2. grammys	0.5549	2. blacklivesmatter	0.3750
	3. bhm	0.4987	3. chapelhillshooting	0.3161
	4. muslimlivesmatter	0.4961	4. jewishlivesmatter	0.2279
	5. alllivesmatter	0.4769	5. whitelivesmatter	0.01764
	6. bluelivesmatter	0.3938	6. ourthreewinners	0.1323
	7. ferguson	0.3226		
	8. icantbreathe	0.2779		
	9. ericgarner	0.1841		
	10. racist	0.1445		
Apr. 4–Apr. 10, 2015	1. walterscott	0.8083	1. blacklivesmatter	1.0000
	2. blacktwitter	0.2307		
	3. icantbreathe	0.1502		
	4. alllivesmatter	0.1488		
	5. ripwalterscott	0.1467		
	6. p2	0.1061		
	7. tcot	0.0645		
	8. kendrickjohnson	0.0645		
	9. ferguson	0.0592		
	10. racism	0.0558		
Apr. 26–May 2, 2015	1. freddiegray	0.4534	1. blacklivesmatter	0.6531
	2. baltimore	0.3441	2. peace	0.3198
	3. blackpower	0.2258	3. baltimoreriots	0.2515
	4. baltimoreriots	0.2120	4. baltimore	0.2289
	5. baltimoreuprising	0.1801	5. justice	0.1321
	6. ferguson	0.1518	6. policelivesmatter	0.1118
	7. solidarity	0.1159	7. freddiegray	0.0975
	8. baltimore	0.1002	8. bluelivesmatter	0.0720
	9. mayday	0.0842	9. baltimoreuprising	0.0540
	10. alllivesmatter	0.0833	10. asianlivesmatter	0.0270
Jun. 17–Jun. 23, 2015	1. charlestonshooting	0.6321	1. charlestonshooting	0.6667
	2. charleston	0.4336	2. blacklivesmatter	0.6238
	3. tcot	0.1727	3. bluelivesmatter	0.4968
	4. confederateflag	0.1547	4. gunsense	0.2571
	5. racheldolezal	0.1168	5. 2a	0.1857
	6. blacktwitter	0.1047	6. pjnet	0.1809
	7. usa	0.0943	7. gohomederay	0.0952
	8. baltimore	0.0904	8. ferguson	0.0952
	9. love	0.0794	9. wakeupamerica	0.0015
	10. staywoke	0.0792	10. tcot	0.0015
Jul. 21–Jul. 27, 2015	1. sandrabland	0.7038	1. blacklivesmatter	0.7842
	2. sayhername	0.1985	2. tcot	0.3689
	3. justiceforsandrabland	0.1533	3. policebrutality	0.2177
	4. blacktwitter	0.1385	4. uniteblue	0.1794
	5. alllivesmatter	0.1154	5. sandrabland	0.1754
	6. wewantanswers	0.1153	6. pjnet	0.1411
	7. blackpower	0.1128	7. justiceforsandrabland	0.1209
	8. sandra	0.0891	8. defundplannedparenthood	0.1189
	9. m4bl	0.0731	9. wakeupamerica	0.1028
	10. blm	0.0656	10. whathappenedtosandrabland	0.0625
