# Supplementary material for: Divergent discourse between protests and counter-protests: #BlackLivesMatter and #AllLivesMatter
Source: PLoS One. 2018 Apr 18;13(4):e0195644. doi: 10.1371/journal.pone.0195644 (PMC5906010; doi:10.1371/journal.pone.0195644)
Supplement: S4 Table — Some #AllLivesMatter topic networks have less than 10 top nodes due to the relatively small size of the networks. (PDF) [file pone.0195644.s023.pdf]

	#BlackLivesMatter		#AllLivesMatter	
	Top Hashtags	PageRank	Top Hashtags	PageRank
Nov. 24–Nov. 30, 2014	1. ferguson	0.2299	1. blacklivesmatter	0.2647
	2. mikebrown	0.0778	2. ferguson	0.2371
	3. fergusondecision	0.0504	3. fergusondecision	0.0575
	4. shutitdown	0.0343	4. mikebrown	0.0509
	5. michaelbrown	0.0258	5. nycprotest	0.0397
	6. blackoutblackfriday	0.0238	6. williamsburg	0.0317
	7. boycottblackfriday	0.0203	7. brownlivesmatter	0.0239
	8. justiceformikebrown	0.0197	8. whitelivesmatter	0.0235
	9. notonedime	0.0176	9. sf	0.0224
	10. america	0.0161	10. blackfridayblackout	0.0210
Dec. 3–Dec. 9, 2014	1. ericgarner	0.1974	1. blacklivesmatter	0.2052
	2. icantbreathe	0.1548	2. icantbreathe	0.1397
	3. ferguson	0.0860	3. ericgarner	0.1295
	4. mikebrown	0.0296	4. ferguson	0.0728
	5. nypd	0.0286	5. tcot	0.0404
	6. shutitdown	0.0251	6. wecantbreathe	0.0379
	7. handsupdontshoot	0.0183	7. mikebrown	0.0371
	8. thisstopstoday	0.0151	8. handsupdontshoot	0.0355
	9. seattle	0.0142	9. rednationrising	0.0268
	10. policebrutality	0.0140	10. shutitdown	0.0267
Dec. 20–Dec. 26, 2014	1. icantbreathe	0.1043	1. blacklivesmatter	0.2042
	2. ferguson	0.0533	2. nypdlivesmatter	0.0955
	3. shutitdown	0.0450	3. nypd	0.0945
	4. antoniomartin	0.0397	4. policelivesmatter	0.0608
	5. ericgarner	0.0333	5. ericgarner	0.0569
	6. nypdlivesmatter	0.0315	6. mikebrown	0.0471
	7. alllivesmatter	0.0256	7. bluelivesmatter	0.0328
	8. moa	0.0253	8. icantbreathe	0.0310
	9. nypd	0.0247	9. nyc	0.0291
	10. mikebrown	0.0227	10. stolenlives	0.0278
Feb. 8–Feb. 14, 2015	1. muslimlivesmatter	0.1418	1. muslimlivesmatter	0.3171
	2. alllivesmatter	0.0730	2. blacklivesmatter	0.1815
	3. handsupdontshoot	0.0686	3. chapelhillshooting	0.1791
	4. grammys	0.0575	4. butinacosmicsensenothingreallymatters	0.0582
	5. mikebrown	0.0485	5. jewishlivesmatter	0.0534
	6. blackhistorymonth	0.0467	6. christianslivesmatter	0.0218
	7. beyhive	0.0447	7. buddhistlivesmatter	0.0218
	8. ferguson	0.0338	8. whitelivesmatter	0.0214
	9. blacktwitter	0.0273	9. rip	0.0197
	10. chapelhillshooting	0.0244	10. hatecrime	0.0181
Apr. 4–Apr. 10, 2015	1. walterscott	0.2141	1. blacklivesmatter	0.4710
	2. ferguson	0.0850	2. walterscott	0.2253
	3. ericgarner	0.0708	3. muslimlivesmatter	0.0705
	4. trayvonmartin	0.0619	4. whitelivesmatter	0.0667
	5. blacktwitter	0.0492	5. icantbreathe	0.0629
	6. icantbreathe	0.0381	6. ripwalterscott	0.0516
	7. mikebrown	0.0231	7. ifnotnowwhen	0.0516
	8. ftp	0.0217		
	9. alllivesmatter	0.0175		
	10. black	0.0148		
Apr. 26–May 2, 2015	1. freddiegray	0.1362	1. blacklivesmatter	0.2104
	2. baltimore	0.1154	2. baltimoreriots	0.1416
	3. baltimoreuprising	0.0850	3. baltimore	0.1115
	4. baltimoreriots	0.0787	4. freddiegray	0.0799
	5. alllivesmatter	0.0153	5. policelivesmatter	0.0427
	6. ericgarner	0.0132	6. baltimoreuprising	0.0419
	7. ferguson	0.0128	7. tcot	0.0327
	8. mayday	0.0115	8. whitelivesmatter	0.0304
	9. blackspring	0.0108	9. wakeupamerica	0.0205
	10. michaelbrown	0.0094	10. prayforbaltimore	0.0192
Jun. 17–Jun. 23, 2015	1. charlestonshooting	0.1670	1. blacklivesmatter	0.2059
	2. racism	0.0361	2. iamame	0.1423
	3. charleston	0.0349	3. wakeupamerica	0.0699
	4. whiteprivilege	0.0217	4. pjnet	0.0656
	5. blacktwitter	0.0216	5. bluelivesmatter	0.0648
	6. ferguson	0.0203	6. tcot	0.0634
	7. itsaracething	0.0198	7. 2a	0.0602
	8. usa	0.0197	8. charlestonshooting	0.0392
	9. southcarolina	0.0189	9. ohhillno	0.0378
	10. alllivesmatter	0.0186	10. cosproject	0.0378
Jul. 21–Jul. 27, 2015	1. sandrabland	0.2013	1. blacklivesmatter	0.2302
	2. sayhername	0.1449	2. sandrabland	0.0996
	3. justiceforsandrabland	0.0422	3. pjnet	0.0661
	4. blackwomenmatter	0.0352	4. defundpp	0.0526
	5. doj	0.0238	5. justiceforsandrabland	0.0505
	6. blacktwitter	0.0201	6. uniteblue	0.0486
	7. unitedblue	0.0169	7. sayhername	0.0472
	8. alllivesmatter	0.0163	8. defundplannedparenthood	0.0470
	9. whathappenedtosandrabland	0.0159	9. tcot	0.0456
	10. tcot	0.0132	10. prolife	0.0344
