# Supplementary material for: Divergent discourse between protests and counter-protests: #BlackLivesMatter and #AllLivesMatter
Source: PLoS One. 2018 Apr 18;13(4):e0195644. doi: 10.1371/journal.pone.0195644 (PMC5906010; doi:10.1371/journal.pone.0195644)
Supplement: S5 Table — The distributions of lexical diversity are statistically significantly different across all time periods. (PDF) [file pone.0195644.s024.pdf]

Time Period	KS Statistic	p-value
November 2014	0.391	1.4×10^{-67}
December 2014	0.481	4.9×10^{-102}
January 2015	0.964	1.0×10^{-308}
February 2015	0.441	7.8×10^{-86}
March 2015	0.772	3.7×10^{-262}
April 2015	0.953	1.0×10^{-308}
May 2015	0.806	1.0×10^{-285}
June 2015	0.845	5.3×10^{-314}
July 2015	0.498	2.4×10^{-109}
August 2015	0.930	1.0×10^{-308}
